# Not So Fast: Strike Kinematics of the Araneoid Trap-Jaw Spider *Pararchaea alba* (Malkaridae: Pararchaeinae)

**DOI:** 10.1093/iob/obab027

**Published:** 2021-10-13

**Authors:** Robert J Kallal, Damian O Elias, Hannah M Wood

**Affiliations:** Department of Entomology, National Museum of Natural History, Smithsonian Institution, Washington, DC, USA; Department of Environmental Science, Policy, and Management, University of California, Berkeley, CA, USA; Department of Entomology, National Museum of Natural History, Smithsonian Institution, Washington, DC, USA

## Abstract

To capture prey otherwise unattainable by muscle function alone, some animal lineages have evolved movements that are driven by stored elastic energy, producing movements of remarkable speed and force. One such example that has evolved multiple times is a trap-jaw mechanism, in which the mouthparts of an animal are loaded with energy as they open to a wide gape and then, when triggered to close, produce a terrific force. Within the spiders (Araneae), this type of attack has thus far solely been documented in the palpimanoid family Mecysmaucheniidae but a similar morphology has also been observed in the distantly related araneoid subfamily Pararchaeinae, leading to speculation of a trap-jaw attack in that lineage as well. Here, using high-speed videography, we test whether cheliceral strike power output suggests elastic-driven movements in the pararchaeine *Pararchaea alba*. The strike speed attained places *P. alba* as a moderately fast striker exceeding the slowest mecysmaucheniids, but failing to the reach the most extreme high-speed strikers that have elastic-driven mechanisms. Using microcomputed tomography, we compare the morphology of *P. alba* chelicerae in the resting and open positions, and their related musculature, and based on results propose a mechanism for cheliceral strike function that includes a torque reversal latching mechanism. Similar to the distantly related trap-jaw mecysmaucheniid spiders, the unusual prosoma morphology in *P. alba* seemingly allows for highly maneuverable chelicerae with a much wider gape than typical spiders, suggesting that increasingly maneuverable joints coupled with a latching mechanism may serve as a precursor to elastic-driven movements.

## Introduction

Animal movement is intrinsically limited due to the force–velocity trade-off, that is, muscles and levers can be maximally optimized for either speed or force, but not both simultaneously. In the case of muscles, the length of the sarcomere dictates muscle performance: muscle fibers with long sarcomeres produce slow and forceful contractions and muscle fibers with short sarcomeres produce fast and low force contractions (Blanco and Patek 2014). This limitation, however, may be overcome by slowly storing energy into elastic structures and then releasing this energy quickly, thereby amplifying power, similar to the operation of a bow and arrow or a catapult. One well-known example of this is the strike of the mantis shrimp (Stomatopoda), in which their powerful raptorial appendage are so fast that they produce cavitation bubbles, producing bursts of heat, light, and sound (Versluis et al. 2000; Patek et al. 2004; Patek et al. 2011). Other arthropod examples are the trap-jaw and snap-jaw attacks found in certain ants (Gronenberg et al. 1993; Larabee et al. 2018) and spiders (Wood et al. 2016; Wood 2020). By triggering specialized hairs (Gronenberg et al. 1993), primed and locked mouthparts are triggered to release and surpass preys’ defenses either by crushing or piecing them.

Within the spiders, an elastic-driven trap-jaw mechanism has been documented in the family Mecysmaucheniidae Simon, 1895, a lineage of small-bodied (∼2–5 mm) spiders known only from New Zealand, and southern Chile and Argentina, within the superfamily Palpimanoidea. These cryptic predators rely on this peculiar attack behavior as they are one of the many spider lineages that do not use sticky silk to intercept their prey, and instead search for it actively. The superfamily as a whole is known for its atypical carapace and cheliceral morphology, including muscle location and function in the modified carapace (Wood and Parkinson 2019). In most spiders, the carapace is a rounded plate that sits on top of the cephalothorax, but within the mecysmaucheniids, the carapace is modified into an elevated, tubular shape that completely encircles the cheliceral bases, which are also elevated and elongated. This morphology and associated internal muscular anatomy have allowed a minority of lineages to evolve an elastic-driven strike mechanism to achieve fast speeds occurring approximately half a thousandth of a second or less (Wood et al. 2016). Not all mecysmaucheniids have this extremely fast strike, however. Within this relatively small family of only 25 described species, average strike speed varies from 0.049 to 8.5 m/s (Wood et al. 2016). In a study focusing on the sister genera *Zearchaea* Wilton, 1946, and *Aotearoa* (Forster and Platnick 1984), the former was three orders of magnitude faster, and was the fastest movement documented to date among arachnids (Wood 2020).

Though the morphology of mecysmaucheniids is specialized, it is not altogether unique. Another lineage of spiders within Araneoidea—the orb-weaving spiders and their relatives—includes a clade that exhibits a similar suite of traits in carapace and chelicerae morphology, the subfamily Pararchaeinae of the family Malkaridae (Forster 1955). The subfamily Pararchaeinae includes 35 species (World Spider Catalog 2021) that have adopted a web-less, active hunter lifestyle, and all species share a similar chelicerae and carapace morphology. Pararchaeinae has a partially overlapping distribution with Mecysmaucheniidae, co-occurring in New Zealand. Similar to the mecysmaucheniids, Pararchaeinae have an elevated, tubular carapace that encircles the cheliceral bases, and the chelicerae are also elevated and elongated (see Supplement S1). In fact, the overall somatic morphology is so similar that these two clades were once considered close relatives (Forster and Platnick 1984). However, multiple recent studies have shown with strong support that these two clades are very distantly related: first, with Sanger data (Rix et al. 2008; Dimitrov et al. 2017) or morphology (Schütt 2000), next, with combined Sanger + morphology (Wood et al. 2012), and most recently with genomic scale data (Fernández et al. 2018; Kulkarni et al. 2020, 2021; Kallal et al. 2021). Despite the peculiar cheliceral/carapace morphology, save for mention of their trap-jaw behaviors (Rix 2006; Wood et al. 2012), and the observation that they feed on collembola (Forster and Platnick 1984), the operation and morphology of their chelicerae have never been examined.

This apparent convergence in carapace and chelicerae shape by Pararchaeinae and Mecysmaucheniidae is exceptional given the morphology of confamilials. Within Malkaridae, there are four subfamilies. One lineage includes Pararchaeinae and Malkarinae, and the other includes Sternoidinae and Tingotinginae (Dimitrov et al. 2017; Hormiga and Scharff 2020). All three non-pararchaeine subfamilies have the more typical spider structure of the prosoma, which features a relatively flat carapace forming a dorsal shield. The situation is similar for mecysmaucheniids; while some close relatives share some of these carapace/chelicerae modifications, as evolutionary relationships become more distant, outgroups have the more typical spider morphology. The distant relationship thus suggests mecysmaucheniids and pararchaeines have convergently evolved this derived carapace. In both clades, the paturon—or basal segment of the chelicera—has evolved long setae on the inner margin that bear a remarkable similarity to the morphology of trigger hairs of trap-jaw ants (Larabee and Suarez 2014).

Wood et al. (2016) hypothesized a suite of traits associated with elastic-driven strikes in mecysmaucheniids, including horizontal cheliceral muscle orientation in a modified prosoma, an intercheliceral sclerite with muscle attachments, a thickened and elongated clypeus, and thick cheliceral ligaments, which have been speculated to serve as the pivot point, anchoring the chelicerae in mecysmaucheniids (Wood et al. 2016; Wood 2020). Within mecysmaucheniids, relative thickness of the clypeus and clypeal muscle ligaments is greater in species with the fastest elastic-driven strikes (Wood et al. 2016; Wood 2020). Mecysmaucheniid species that have slower, seemingly muscle-driven strikes have a greater number of muscles associated with the chelicerae—namely the anterior medial inner and outer muscles and the endosternite muscle—which are present in the slower, muscle-driven lineage but not in faster, elastic-driven lineages, where closure is driven by elastics. While the distantly related Mecysmaucheniidae and Pararchaeinae share an overall similar morphology of the carapace and chelicerae, we do not yet understand whether cheliceral function and internal musculature is similar as well.

Previous research on elastic-driven movements focused on documenting whether a biological movement had a greater power output than was possible by muscle alone. If so, this was considered indirect evidence that an elastic mechanism was present and that “power-amplification” occurred, a term commonly used to describe these systems (e.g., Peplowski and Marsh 1997; Aerts 1998; Henry et al. 2005; Astley and Roberts 2011; Claverie et al. 2011; Wood et al. 2016; Larabee et al. 2017, 2018; Freymiller et al. 2019). Under these criteria, 400 watt kg^–1^ is typically considered to be the maximum power output of conventional muscle (Askew and Marsh 2002). A latch-mediated spring actuation (LaMSA) framework requires springs to store energy and latches to release it at levels higher than conventional muscles, and should be present in systems exhibiting power amplification (Longo et al. 2019).

Here, for the first time, we analyze the strike kinematics and document the morphology of the araneoid trap-jaw spider *Pararchaea alba* through the lens of LaMSA, specifically testing whether the power output of the cheliceral strike is elevated. In order to examine how *P. alba* fits into the landscape of trap-jaw spider kinematics, we use high-speed videography to quantify angular velocity and acceleration, and to estimate strike power output. Then, using microcomputed tomography (micro-CT), we scanned specimens with chelicerae in the resting and open positions to characterize the positions of muscles and the associated intercheliceral sclerite. Together, these are synthesized in a framework to determine the latch and spring anatomy in this unique spider.

## Methods and materials

### High-speed videography

Eleven specimens—9 females and 2 males—were measured and recorded. Specimens were collected in 2011 (New Zealand, Canterbury Region, near Lewis Pass) by sifting moss on the forest floor, on logs and at the base of tree trunks. For this method, moss was pulled up and placed in a litter sifter: a long bag with handles at the top, wire mesh in the middle, and a bottom that could be tied closed. The handles were shaken and small particles fall through the wire mesh, and concentrated leaf litter is collected at the bottom. This material was then spread out on a white sheet and specimens were collected alive when they began to move.

For video recording, spiders, unconstrained within glass vials, were filmed with the camera oriented perpendicular to the plane of cheliceral movement (Supplement S2). Recordings were conducted at 1000–10,000 frames per second, with resolutions of 128 × 48, 512 × 128, and 1024 × 1024, using Photron cameras (SA3 and a FastCam Mini AX200), Photron USA Inc., San Diego, USA). A strike was elicited by rubbing the anterior of the chelicerae with an eyelash attached to a pin. Due to the lunging motion of *P. alba* while striking, we used the “Template Matching and Slice Alignment” plugin (Tseng et al. 2011) (code: https://github.com/qztseng/imagej_plugins) in FIJI (Schindelin et al. 2012) to isolate only cheliceral rotational movement. To measure kinematic variables, using the “point tool” in FIJI, the distal anteriomesal cheliceral edge was digitally tracked over the course of cheliceral closure. Due to the high ratio of frames per second to movement, points were digitized at set intervals rather than on every frame from open position to closed position (e.g., one point per four images). These data are available in Supplement S3. Using the “Pspline” function (Ramsey and Ripley 2017) in the “survival” R-package (Therneau and Grambsch 2000; Therneau 2015) following Larabee et al. (2018) and Wood (2020), the cumulative cheliceral displacement was fit with a quintic spline (Supplement S4). Angular velocity and acceleration were calculated as the 1st and 2nd derivatives. Linear velocity and acceleration were calculated from the arc length traveled by the distal tracked point on each chelicera. Treating each chelicera as a thin rod of uniform density that rotates around a fixed point, the following calculations were made. The moment of inertia, *I*, was calculated as $I\ = \ \frac{1}{3}M{L^2}$, where *M* and *L* are the chelicera mass and length. The kinetic energy of the chelicera, *E*, was calculated as $E\ = \ \frac{1}{2}I{\omega ^2}.$ The strike power, *P*, was calculated as $P\ = \ \frac{{{E_{max}}}}{{{t_{E,\ max}}}},,\ $where *E_max_* is the maximum energy of the of the chelicera and *t_E_,_max_* is the time when *E* is at the maximum. Power output, *O*, of the cheliceral strike is $O\ = \ \frac{P}{m}$, where *m* is the adductor muscle mass. Time was based on the frame rate used for the video recording. Cheliceral muscle masses were estimated based on cheliceral length and data from previously measured palpimanoid trap-jaw spiders (Wood et al. 2016). After video recording was complete, specimens were preserved in 75% ethanol. Cheliceral length measurements were then collected from these preserved specimens using a Leica M205 C microscope (Leica Microsystems Inc., Buffalo Grove, USA).

### Microcomputed tomography

Two CT scans were performed on *P*. *alba*, one on a specimen with the chelicerae in the closed, resting position (CASENT9034335), and one on a specimen with the chelicerae in the open position (CASENT9034320). Specimens were collected as above and were preserved in 75% ethanol. Ethanol acts as a fixative, preserving tissue through dehydration; however, at lower concentrations, ethanol preserves tissue without apparent shriveling or distortion, allowing for morphological study. For this reason, in natural history collections, spider specimens are typically preserved and stored in ethanol at concentrations of 70–80%, and soft structures (such as muscles, glands, and ganglia) retain much of the original shape as when alive.

Prior to scanning, specimens were stained overnight in Lugol's solution, and then were washed in ethanol for around 30 min just before scanning. Specimens were then scanned in ethanol. One scan was conducted at Lawrence Berkeley National Lab Advanced Light Source synchrotron, Berkeley, CA, on Beamline 8.3.2 (hard X-ray microtomography) with scan parameters following Wood and Parkinson (2019): scan energy of 33.5 kV and a voxel size of 1.3 μm. The other scan was performed at the National Museum of Natural History, Smithsonian Institution, Washington, DC, on a GE Phoenix nano-CT instrument (General Electric Company, Boston, USA): scanning at 100 kV and 131 uA; four half second projections were averaged, with a total of 3000 projections analyzed; voxel size of 1.45 μm, and magnification of 137.9. Three-dimensional reconstructions were produced from these scans using VG Studio Max version 3.2 (Volume Graphics, Hexagon AB, Stockholm, Sweden) and were processed using Avizo Lite version 2019.1 (Thermo Scientific, Hampton, USA). The carapace, paturon, fang, intercheliceral sclerite, and muscles and ligaments associated with the paturon were segmented, resulting in a 3D digital model of these structures.

The following measurements were taken from the surface meshes derived from the segmentations using the measurement tool in Avizo Lite, for both CT scans: clypeus thickness (measured near medial prominence), and for all muscles associated with the cheliceral bases, tendon lengths, and fiber lengths (shortest and longest). In the open specimen, the anterior median inner and posterior median muscles were pulled away from the carapace and length measurements were extrapolated to the inner margin of the carapace. Muscle measurements were taken for both the left and right side and reported as averages. Clypeus thickness was reduced to a ratio of clypeus thickness to the carapace thickness between the anterior median eyes following Wood (2020). Muscle tendon and fiber lengths were size corrected by carapace width to make measurements comparable between specimens/scans.

## Results

**Fig. 1:**
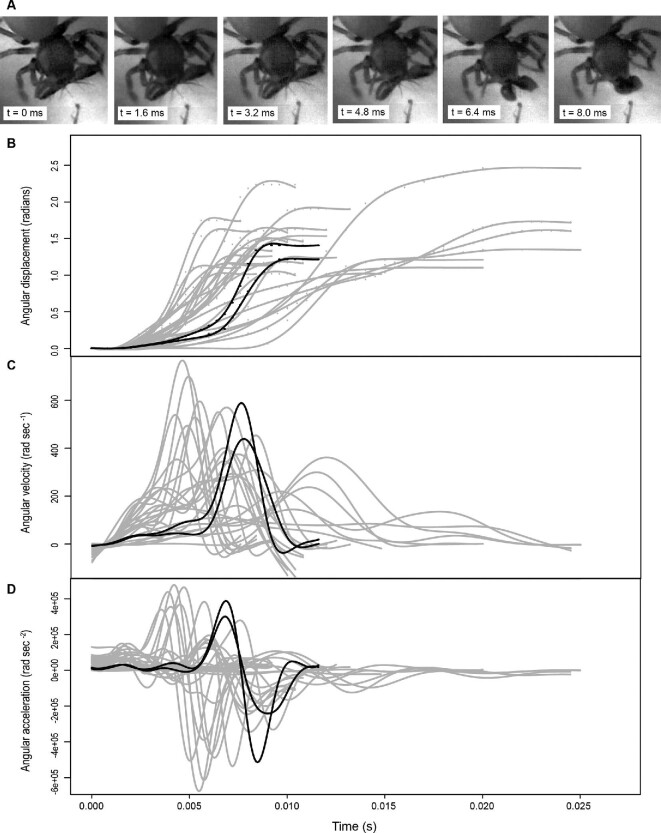
High-speed images and related kinematic data. (**A**) Video frames associated with individual 16 (CASENT9034331), video A, recorded at 10,000 frames per second, with 0.016 s between depicted frames. Kinematics, with video A, from individual 16, in black and all other strikes in gray. **(B)** Displacement of chelicerae during strikes, with raw data shown as points and fitted splines as lines. **(C)** Fitted splines for velocity. **(D)** Fitted splines for acceleration.

A total of 27 strikes were recorded for 11 individuals. Of these, cheliceral lengths ranged from 0.43 to 0.48 mm, with an average of 0.46 ± 0.019 mm (*n* = 11). Cheliceral gape was 172.4 ± 7.9° (mean ± standard deviation), ranging from 152.62° to 179.78° (*n* = 20). The average strike duration was 0.012 ± 0.009 s, ranging from 0.0034 to 0.0363 s (*n* = 27). Average opening duration from the closed to reset position was 0.054 ± 0.02 s (*n* = 12), ranging from 0.039 to 0.112 s. The chelicerae were always observed to move synchronously (moving at the same time) when unobstructed. While the left and right chelicera may have different resulting curves in a given strike, it is presumed this is a result of camera angle. Of the total videos, kinematic calculations were performed on a subset of 16 high-quality video strikes from 10 individuals (Fig. 1). Kinematic results were averaged between synchronously striking chelicerae, then all strikes’ parameters were averaged for a value for the species. Peak angular velocities were 384.25 ± 151.64 rad s^–1^ (126.93–653.04 rad s^–1^) and peak angular accelerations were 19.640 ± 13.487 × 10^4^ rad s^–2^ (2.772–41.626 × 10^4^ rad s^–2^). Peak linear velocities were 0.177 ± 0.072 m s^–1^ (0.057–0.300 m s^–1^) and peak linear accelerations were 90.92 ± 63.43 m s^–2^ (12.47–199.764 m s^–2^). The mass-specific power output was 1.95 ± 1.90 watt kg ^–1^ (0.12–6.81 watt kg ^–1^). Strike information is summarized in Supplement S5.

**Fig. 2:**
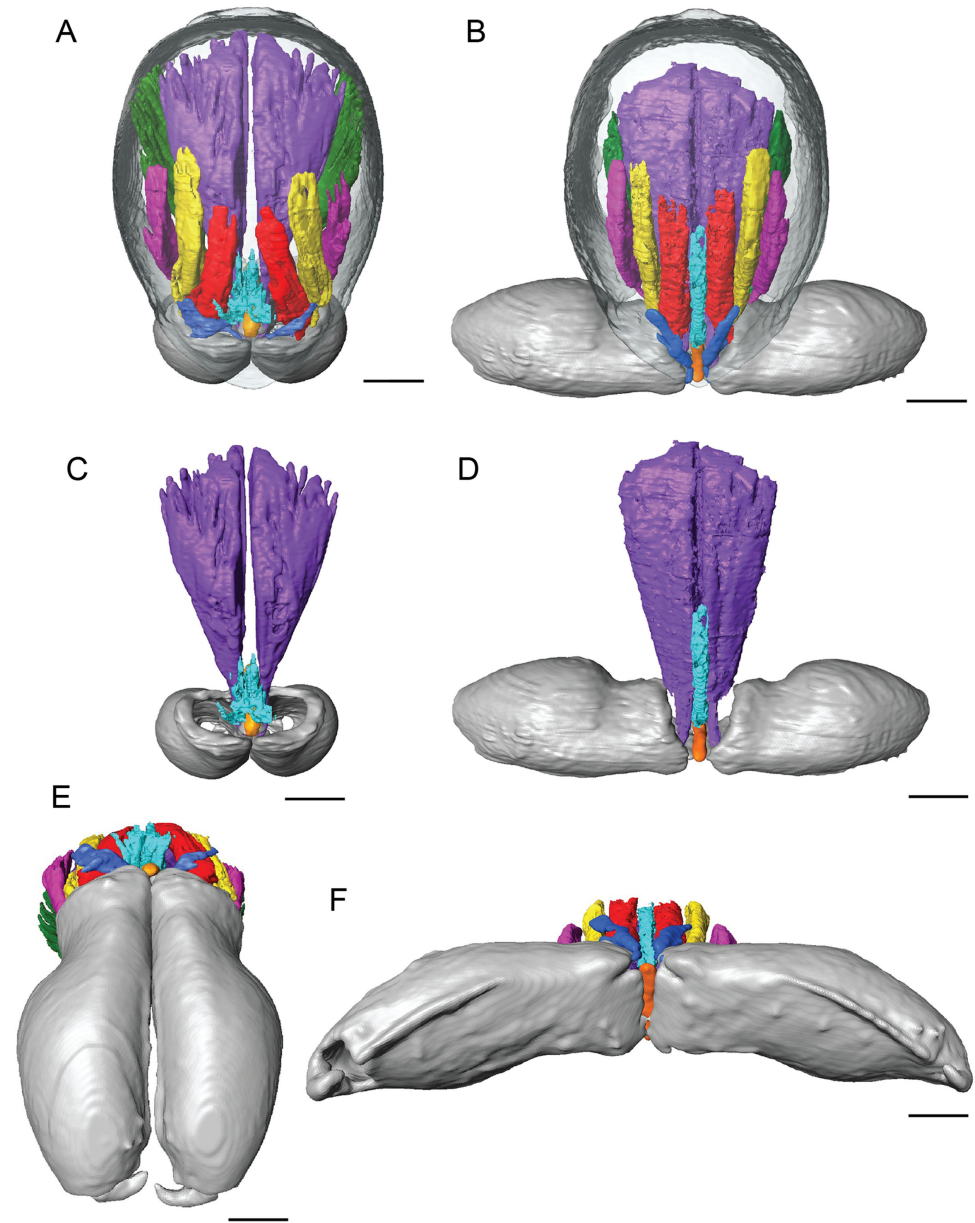
Images of micro-CT scans with different sets of cheliceral muscles digitally segmented. Chelicerae are closed in the left column and open in the right. (**A, B**) Dorsal. (**C, D**) Frontal. Color codes: purple, anterior median inner; blue, anterior median outer; red, anterior outer; yellow, lateral anterior; magenta, lateral posterior; green, posterior median; aqua, intercheliceral. Scale bars: 0.1 mm.

**Fig. 3:**
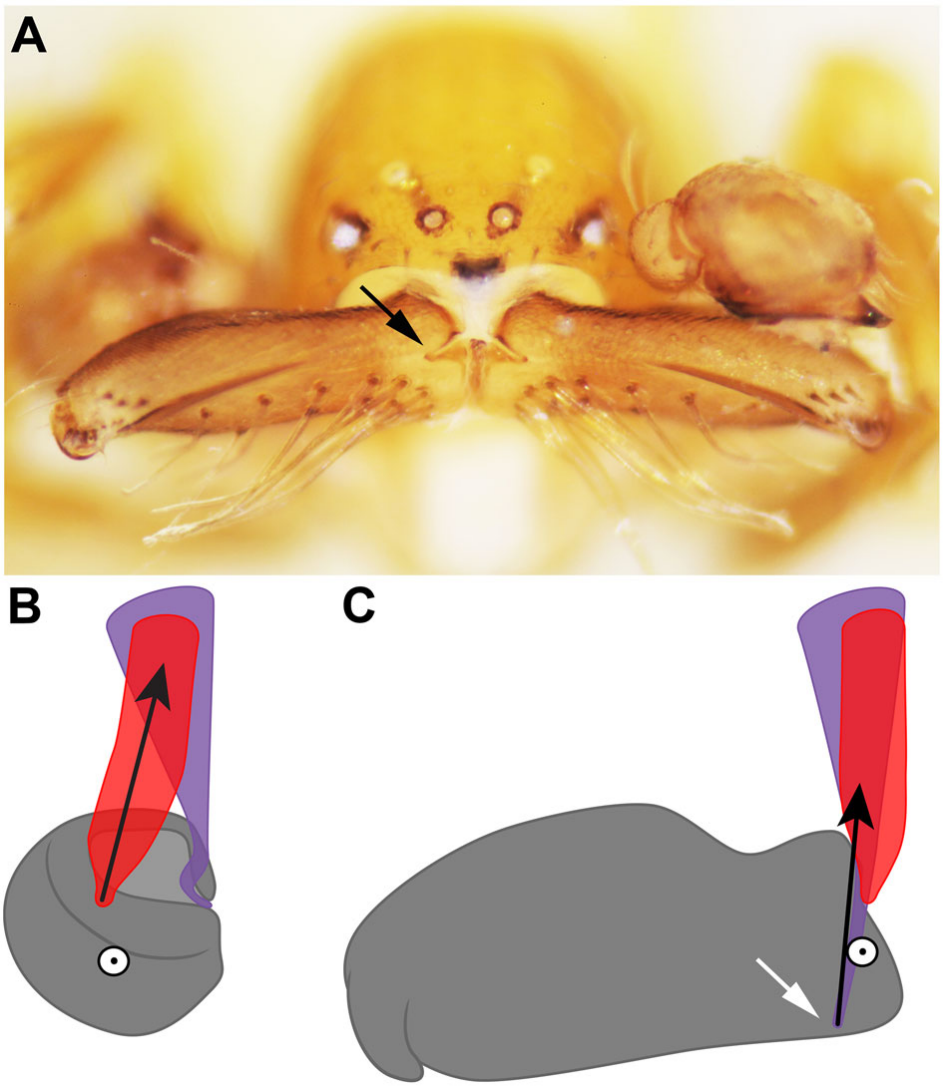
The proposed torque reversal mechanism. **(A)***Pararchaea alba*, male (CASENT9034332), anterior view, specimen preserved with chelicerae open, black arrow showing the inner cheliceral groove where the closure muscle (color coded purple in figures) inserts. **(B, C)** Illustration of the torque reversal mechanism, dorsal view, right chelicera; black arrows represent the direction of muscle pull; white circle with black outline represents the pivot point. In panel (B), the chelicera is at rest and the closure muscle (color coded purple) is more medially located than the pivot point. Contraction of the abductor muscle (color coded red), represented by the black arrow, causes the chelicera to move to the open position, illustrated in panel (C). In the open position, the closure muscle insertion (white arrow), due to the cheliceral groove (black arrow in A), now is slightly more laterally located than the pivot point, and contraction of the closure muscle in this position may hold the chelicera open. Contraction of the ICS muscle (not shown, but color coded aqua in other figures) would pull the chelicera closed, swinging the closure muscle back to being on the inner side of the pivot point, allowing the chelicera to close.

For the two 3D reconstructions produced from the CT scans, clypeus thickness was 1.26 ± 0.24 μm (*n* = 2). Tendon and muscle fiber lengths are summarized in Table 1. Endosternite muscles (color coded orange from Wood and Parkinson 2019) were not observed in *P*. *alba*. The clypeal ligaments that were observed in mecysmaucheniid species were not observed in *P. alba* (Fig. 2), but instead thickened, semi-sclerotized quasi-ligaments may serve the same purpose of constricting movement, so that the chelicerae pivot occurs in the anterior–lateral corner of the cheliceral base, and may contribute to the strike mechanism (Fig. 3).

**Table 1:** Cheliceral muscle measurements of *P*. *alba*, averaged between left and right pairs, for the chelicerae in the open position prior to a strike and in the closed or resting position. Raw measurements are in microns, with size-corrected measurements (by carapace width) in parentheses (500.4 and 495.5 μm for closed and open chelicerae specimens, respectively). Muscle lengths with asterisks are extrapolated to interior surface of carapace

	Tendon, closed	Tendon, open	Shortest fiber, closed	Shortest fiber, open	Longest fiber, closed	Longest fiber, open
Anterior medial inner	140.04 (0.27)	156.58 (0.31)	185.18 (0.37)	399.52^*^ (0.81^*^)	370.45 (0.74)	522.22^*^ (1.05^*^)
Anterior median outer	20.73 (0.04)	21.12 (0.05)	100.52 (0.20)	130.01 (0.26)	105.46 (0.21)	154.42 (0.31)
Anterior outer	29.69 (0.06)	28.66 (0.06)	113.01 (0.23)	236.06 (0.48)	235.38 (0.47)	319.53 (0.64)
Lateral anterior	33.31 (0.07)	33.67 (0.07)	143.12 (0.29)	150.00 (0.30)	258.94 (0.52)	311.41 (0.63)
Lateral posterior	45.07 (0.09)	40.33 (0.08)	129.79 (0.26)	123.20 (0.25)	230.41 (0.46)	265.74 (0.54)
Posterior median	91.43 (0.18)	64.17 (0.13)	199.25 (0.40)	330.87^*^ (0.67^*^)	312.83 (0.63)	396.70^*^ (0.80^*^)
Intercheliceral sclerite	17.79 (0.04)	30.48 (0.06)	103.13 (0.21)	86.21 (0.17)	110.87 (0.22)	288.48 (0.58)

## Discussion

### Kinematics

Previous research on elastic-driven movements focused on documenting whether a biological movement had a greater power output than was possible by muscle alone. If so, this was considered indirect evidence that an elastic mechanism was present and the term “power-amplification” was used to describe these systems (e.g., Peplowski and Marsh 1997; Aerts 1998; Henry et al. 2005; Astley and Roberts 2011; Claverie et al. 2011; Wood et al. 2016; Larabee et al. 2017, 2018; Freymiller et al. 2019). Under these criteria and based on the 400 watt kg^–1^ maximum power output of conventional muscle (Askew and Marsh 2002), the 1.95 ± 1.90 watt kg ^–1^ strike of *P*. *alba* (Fig. 1) fails to exceed this threshold. Thus, *P*. *alba* is by no means exceptional when it comes to extremely fast movements. However, applying this “power amplification” criterion can result in overlooking elastic mechanisms that produce unexceptional movements (Longo et al. 2019). Alternatively, LaMSA has been promoted as a replacement for the term “power amplification” with the idea that future research should be reframed from focusing on the limitations of muscle power, to instead focusing on the springs that actuate movement and the latches that control how elastic energy is released (Longo et al. 2019).

Under LaMSA criteria, the speed and morphology of *P. alba* bears special consideration. In the greater spider tree of life, although some intermediates exist, the only lineages with comparable carapace and chelicera morphology and associated trap-jaw predatory behaviors are the pararchaeine malkarids and the mecysmaucheniids. These two clades shared a recent common ancestor nearly 250 million years ago (Magalhães et al. 2020; Kallal et al. 2021). To appreciate the evolutionary distance separating these two clades, see figures 2 and 3 of Kallal et al. (2021): mecysmaucheniids are in Palpimanoidea on figure 2a and Pararchaeinae are within Malkaridae in Araneoidea on figure 3a. Pararchaeinae is approximately 90 million years removed from its closest relative, whereas Mecysmaucheniidae is approximately 200 million years removed from its sister lineage. Nevertheless, *P*. *alba* has multiple features convergent with mecysmaucheniids: the combined suite of the carapace shape, cheliceral shape, muscle orientation, and long setae that project forward when the chelicerae are open observed in these taxa are apparent convergent adaptations in strike performance.

The morphological similarities to mecysmaucheniids lend themselves to a number of functional similarities as well. *Pararchaea alba* holds its chelicerae open prior to a strike, has highly maneuverable chelicerae with a wide gape, and has a strike speed that is somewhat elevated. In a previous similar study, the mecysmaucheniids *Aotearoa* and *Zearchaea* sister genera were examined, with the latter having the fastest elastic-driven movements ever documented among arachnids (spiders, scorpions, mites, and their relatives; Wood 2020). *Aotearoa*, on the other hand, had slower strikes that may not involve elastic energy storage. Similar to the slower striking *Aotearoa*, the anterior medial muscles are present in *Pararchaea* but like *Zearchaea*, cheliceral gape approaches 180°; *Aotearoa*’s average gape is approximately half of that (Wood 2020). While *P. alba* achieves 1.5% the peak angular velocity of *Zearchaea* sp. peak angular velocity (*P. alba* = 0.384 ± 0.151 × 10^3^ rad s^–1^; *Zearchaea* = 25.0 ± 4.8 × 10^3^ rad s^–1^), it is faster than *Aotearoa* with a peak angular velocity of 0.0648 ± 0.022 rad s^–1^. This is notable because *P*. *alba*, with a body length of less than 3 mm (Rix 2006), is smaller than *Aotearoa* (body length ∼3 mm; Forster and Platnick 1984), but still capable of achieving a faster cheliceral strike. Thus, *P. alba* does not have the extreme record-breaking strikes of *Zearchaea*, yet, strike speed is elevated and an elastic mechanism may be involved. The morphology of *P. alba* allows it to have a much wider gape than typical spiders (nearly 180°) due to more maneuverable chelicerae. Maneuverable joints coupled with a latching mechanism may be a precursor to extreme elastic-driven movements, consistent with similar elastic-driven mechanisms in other lineages (Kaji et al. 2018; Booher et al. 2021).

Very little is known about the prey choice of trap-jaw spiders in both Malkaridae and Mecysmaucheniidae. Neither lineage builds a web to passively capture prey, but instead are active hunters. Mecysmaucheniids have a handful of field observations where they were captured with other spiders in their chelicerae (Wood et al. 2012), but in the lab they are generalists and will feed on *Drosophila* or moths (Vellard 1957; Forster and Platnick 1984). These observations are likely for the larger mecysmaucheniid species, which also are the species with the slower cheliceral strikes (Wood et al. 2016; Wood 2020). The smaller species of mecysmaucheniids (with a body length approaching around 2 mm), which also have the fastest elastic-driven strikes, have been observed a handful of times feeding on collembola in a laboratory setting (Wood et al. 2012), but observations are so scarce for these cryptic spiders that much more research is needed. In captivity, pararchaeinaes have been observed to feed on collembola, which are found in similar habitats as trap-jaw spiders (Forster and Platnick 1984). Colloquially called springtails, these animals are known for their predator-evading leaps. The leap take-off velocity of a globular springtail, *Bourletiella hortensis*, was recorded at about 2 m s^–1^ (Sudo et al. 2013), which far exceeds *Pararchaea*’s average peak linear strike velocity of 0.177 m s^–1^. However, strike duration, escape duration, and even loading duration of the escape jump are probably more important than velocity in determining whether *Pararchaea* can capture a springtail prey. For *B. hortensis*, furcula contact time is 1.12 ms, and the time from take-off to reach maximum height is 0.15 s (Sudo et al. 2013). For *P. alba*, average strike duration is 0.017 s, which may allow for prey capture if there is a long loading time for the springtail (unknown), or if capture is made during the springtail's ascent. Yet, at this time we do not know what species of springtail *P. alba* preys on, if any, and thus, more research is needed. Trap-jaw mechanisms not only provide speed but also the possibility of piercing tough prey or capturing larger prey, but the details remain speculative.

### Hypothesized mechanism

Based on results from CT scans and kinematic analysis, we propose a torque reversal mechanism for the cheliceral strike in *P*. *alba*. The closure muscle (color coded purple in Figs. 2 and 3) attaches to the cheliceral bases in a novel way in Pararchaeinae. In most spiders, the muscles that operate the cheliceral bases attach to the basal edge of the cheliceral base, but the CT scan of *P. alba* revealed that the closure muscle (color coded purple) insertion is in a more distal position due to a narrow groove in the inner margin of the cheliceral base cuticle (Fig. 3). This novel position is hypothesized to create a torque reversal mechanism whereby the closure muscles help to hold the chelicerae open when the gape is wide, but pull the chelicerae closed when the gape decreases. Reverse torque mechanisms have been documented in snapping shrimp, which have elastic-driven movements (Kaji et al. 2018). In *P*. *alba*, muscles do not have different functional regions as in snapping shrimp, and instead a separate closure muscle (intercheliceral sclerite [ICS] muscle color coded aqua) pulls the ICS back and the cheliceral bases along with it, which changes the sign of the moment arm as the closure muscle (color coded purple) switches from being exterior to being interior to the pivot point (Fig. 3).

Our hypothesis for the mechanism of the cheliceral strike is as follows: to open the chelicerae, the anterior outer muscles (color coded red) contract, rotating the cheliceral bases and intercheliceral sclerite forward and creating a wide gape of nearly 180° (Fig. 2). As the chelicerae go from closed to open, the line of pull from the closure muscles (color coded purple) moves from an interior position relative to the pivot point to an exterior position, reversing the torque. We hypothesize that a thickening of one section of the membranous cuticle that surrounds the cheliceral bases acts as a ligament to stabilize the chelicerae so that they rotate around the anterior lateral corner (Fig. 3). The high-speed videos revealed that the chelicerae close in unison. We hypothesize that contraction of the muscles associated with the ICS (color coded aqua) pulls the ICS back along with the cheliceral bases, releasing the chelicerae to strike. The ICS muscle (color coded aqua) is oriented more perpendicularly to the plane of cheliceral closure movement, and may destabilize the system by closing the chelicerae enough so that the closure muscles (color coded purple) move to the interior position relative to the pivot point (Fig. 3), reversing the torque. This study did not address whether there are elastic elements present in the cheliceral strike mechanism, but future study should examine whether the closure muscles (color coded purple) have elastic elements in the tendons or whether another unidentified elastic element is present.

Other results from this study include indirect evidence for hydraulic pressure contributing to cheliceral abduction. When comparing size-corrected cheliceral muscle lengths from CT scans of the specimen with closed versus the specimen with open chelicerae—a novel comparison due to the rarity of specimens of trap-jaw spiders preserved in the open position—we did not observe shorter muscles in the open chelicerae specimen (Table 1; Fig. 2). In the open position, one would expect the distance to be shorter between where the abduction muscle inserts on the carapace and chelicerae. Instead, the distances of all muscles between insertion points increased (exception in the shortest fibers of the ICS muscle). This suggests muscles are not wholly responsible for the extension of *Pararchaea*’s chelicerae into their open position. Hydraulic pressure is a possible alternative and one that is already found in spiders. For instance, spiders use pressurized hemolymph to extend (but not contract) their legs, and most male spiders operate their pedipalps—intromittent sexual organs composed of sclerites, membranes, and hematodocha—in a similar way (Manton 1958; Foelix 2011; Kropf 2013). It has been suggested that hydraulics are involved in the high-speed expulsion of silk and venom in spitting spider chelicerae (Millot 1949) and also in mecysmaucheniids (Wood 2020); however, to date no studies have directly tested for this.

### Functional versatility

A possible rationale for moderate speeds and partially optimized morphology could be selection for versatility. While *Pararchaea* may lack extreme speed, it may have a more flexible striking ability. Analysis of strike kinematics shows that *P. alba* shows a range of durations that are an order of magnitude different (0.0034–0.0363 s) than the fastest peak angular velocities five times greater than the slowest (126.93–653.04 rad s^–1^) (Fig. 2). Unfortunately, we know so little about cheliceral mechanics across spiders that there is no baseline for comparison. We observed a broader range of durations, linear and angular velocities, linear and angular accelerations, and power in *P. alba* than in comparable work on two mecysmaucheniid species, one seemingly with slower muscle-driven strikes and one with fast elastic-driven strikes (Wood 2020). While this may be an artifact of sampling, it may also be an indicator of strike variability.

Furthermore, while chelicerae have a major function in catching prey in trap-jaw species, that is not their sole purpose. The chelicerae are multitasking structures that are crucial to spider survival and biology. Spider chelicerae tasks include venom delivery and handling seized prey as well as non-foraging functions such as copulation, communication, preening, defense, and egg sac manipulation. This is to say that like all spiders, the form and function of the chelicerae of *P. alba* are being shaped by a variety of forces and trade-offs—in this case, specialization into an extremely fast strike may come with a cost of losing other functions, such as envenomation or gripping. Unlike the simple movements in one plane of many arthropod joints, this joint that operates the cheliceral bases in spiders has at least nine different muscles and multiple degrees of movements. The complexity of this joint may allow for a variety of tasks to be performed. In mecysmaucheniids, the lineage with the fastest documented elastic-driven strike, *Zearchaea*, has lost three sets of cheliceral muscles compared with its closest relative (Wood 2020). That is, there may be a trade-off between specialization and versatility.

## Conclusion

*Pararchaea alba* is an intriguing lineage that appears to have evolved a torque reversal latching mechanism, possibly with some elastic energy storage, and moderately elevated striking speeds. The highly modified prosoma is remarkably convergent to the distantly related trap-jaw mecysmaucheniid spiders. It remains to be seen how other pararchaeines compare with *P. alba* and mecysmaucheniids, much less other spiders with less specialized cheliceral functions. Yet, as more future studies are done, we can begin to understand diversification of form and function in spider chelicerae in a new light. Although these strike kinematics do not shatter records of the fastest and most similar species, *P. alba* can be used in future comparative studies to document why and how elastic-driven mechanisms evolve. The unusual prosoma morphology suggests this lineage is an intermediate to extreme elastic-driven movements; it has maneuverable chelicerae with a much wider gape than typical spiders, including with a possible torque reversal latch, and this may contribute to the observed elevated cheliceral speeds.

## Supplementary Material

obab027_Supplemental_Files
