# Supplementary material for: Not So Fast: Strike Kinematics of the Araneoid Trap-Jaw Spider Pararchaea alba (Malkaridae: Pararchaeinae)
Source: Integr Org Biol. 2021 Oct 13;3(1):obab027. doi: 10.1093/iob/obab027 (PMC8514421; doi:10.1093/iob/obab027)
Supplement: obab027_Supplemental_Files [file obab027_supplemental_files.zip › marathi abstract.docx]

एकट्या स्नायुंच्या कार्याद्वारे अप्राप्य शिकार पकडण्यासाठी काही प्राण्यांच्या वंशामध्ये विशिष्ट हालचाली विकसित झाल्या आहेत, ज्या संचयीत लवचिक ऊर्जे द्वारे उल्लेखनीय वेग आणि शक्तीशाली हालचाली निर्माण करतात.या हालचालींचे असे एक उदाहरण ज्या मध्ये अनेकदा उत्क्रांती झाली आहे ते म्हणजे सापळा-जबडा यंत्रणा, या यंत्रणेमध्ये प्राण्याच्या जबड्यात (in chelicerae) अंतर निर्माण केले जाते ज्या मध्ये भरपूर ऊर्जा सामावलेली असते आणि जेव्हा ते बंद होण्यास सुरुवात होते तेव्हा प्रचंड शक्ती निर्माण होते.  कोळ्यांच्या प्रजातींमध्ये अश्या प्रकारच्या हालचालींचे लेखी पुरावे फक्त Palpimanoidea superfamily मधील Mecysmaucheniidae मध्ये आढळतात, परंतु त्याच्याशी साधर्म्य असणारे अशाच स्वरूपाचे काही हालचालींचे प्रकार दुरुन संबंधित असलेल्या Araneoidea subfamily Pararchaeinae मध्ये देखील आढळले आहेत. ज्यामुळे त्या वंशामध्ये देखील सापळा-जबडा यंत्रणेचा वापर करून हल्ला करण्याची क्षमता आहे असा तर्क केला जातो. येथे high-speed videography वापरून, Pararchaeinae *Pararchaea alba*मध्ये जबड्याची हल्ला करण्याची शक्ती ही लवचिक ऊर्जे द्वारे होणारी हालचाल आहे का? हे आम्ही तपासून पाहिले. *P. alba*  मध्ये  जबड्याने हल्ला करण्याची गती मध्यम वेग असणारी आहे तर Mecysmaucheniids मध्ये गती मंदावते, परंतु लवचिकता असणाऱ्या स्नायूंची यंत्रणा वापरून वेगवान  गतीने हि क्रिया करणाऱ्या कोळ्यांपर्यंत पोहोचण्यात हि पद्धत अयशस्वी आहे. Micro-computed-Tomography वापरून आम्ही *P. alba*च्या जबड्याची विश्रांतीच्या आणि हल्ला करण्याच्या स्थितीतील रूपात्मक रचनेची तुलना केली. यामध्ये असे आढळून आले की  जबड्याची व त्यांचे संबंधित स्नायू हल्ला करण्याच्या कार्यात हि यंत्रणा वापरतात, त्याचबरोबरीने  जबड्याची खुल्या स्थितीत अडकवण्याची क्रिया देखील समाविष्ट आहे. त्यांच्याशी दुरून संबंधित असलेल्या Mecysmaucheniidae कोळ्यांप्रमाणे, *P.alba*मध्ये असामान्य असा prosoma (शरीराचा डोके, डोळे, छाती व पाय असणारा भाग) हा भाग त्याच्या रूपात्मक रचनेमुळे हाताळण्यायोग्य असणारे जबडे वापरून त्यामध्ये जास्तीत जास्त अंतर निर्माण करण्यासाठी वापरला जातो, यामधून हे सूचित होते की, अत्यंत कुशल असणारे हे सांधे खुल्या स्थितीत अडकवण्यासाठी लवचिकता असणाऱ्या स्नायूंची यंत्रणा अग्रदूत म्हणून भूमिका बजावते.
