# Supplementary material for: Not So Fast: Strike Kinematics of the Araneoid Trap-Jaw Spider Pararchaea alba (Malkaridae: Pararchaeinae)
Source: Integr Org Biol. 2021 Oct 13;3(1):obab027. doi: 10.1093/iob/obab027 (PMC8514421; doi:10.1093/iob/obab027)
Supplement: obab027_Supplemental_Files [file obab027_supplemental_files.zip › portuguese abstract.docx]

Para capturar presas impossíveis de apanhar apenas com função muscular, algumas linhagens animais desenvolveram movimentos que são impulsionados pela energia elástica armazenada, produzindo movimentos de velocidade e força notáveis. Um exemplo que evoluiu múltiplas vezes é um mecanismo de mandíbula-armadilha, no qual as peças bucais de um animal são carregadas com energia quando se abrem, e então, quando provocadas para fechar, produzem uma força incrível. Dentro das aranhas (Araneae), este tipo de ataque foi até agora apenas documentado na família palpimanóide Mecysmaucheniidae, mas uma morfologia semelhante também foi observada na distantemente relacionada subfamília araneóide Pararchaeinae, levando à especulação de um ataque de mandíbula-armadilha também nessa linhagem. Aqui, usando videografia de alta velocidade, testamos se o produto da potência de ataque queliceral sugere movimentos elásticos na Pararqueína *Pararchaea alba*. A velocidade de ataque obtida coloca *P. alba* como um atacante moderadamente rápido, excedendo os mecysmaucheniídeos mais lentos, mas não alcançando os atacantes de alta velocidade mais extremos que têm mecanismos acionados por elásticos. Usando micro-tomografia computadorizada, comparamos a morfologia das quelíceras de *P. alba* nas posições de repouso e abertas, e sua musculatura relacionada, e com base nos resultados propomos um mecanismo para a função de ataque queliceral que inclui um mecanismo de travamento de reversão de torque. Semelhante às distantemente relacionadas aranhas mecysmaucheniídeas de mandíbula-armadilha, a morfologia incomum do prosoma em *P. alba* aparentemente permite quelíceras altamente manobráveis ​​com uma abertura muito maior do que as aranhas típicas, sugerindo que juntas cada vez mais manobráveis ​​acopladas a um mecanismo de travamento podem servir como um precursor para movimentos acionados por elásticos.
