# Supplementary material for: Not So Fast: Strike Kinematics of the Araneoid Trap-Jaw Spider Pararchaea alba (Malkaridae: Pararchaeinae)
Source: Integr Org Biol. 2021 Oct 13;3(1):obab027. doi: 10.1093/iob/obab027 (PMC8514421; doi:10.1093/iob/obab027)
Supplement: obab027_Supplemental_Files [file obab027_supplemental_files.zip › spanish abstract.docx]

Para capturar presas que de otro modo serían inalcanzables únicamente por la función muscular, algunos linajes de animales han desarrollado movimientos que son impulsados ​​por energía elástica almacenada, produciendo movimientos de notable velocidad y fuerza. Uno de esos ejemplos que ha evolucionado varias veces es un mecanismo de mandíbula trampa, en el que las piezas bucales de un animal se cargan con energía cuando se abren con gran abertura y luego, cuando se disparan para cerrarse, produciendo una fuerza tremenda. Dentro de las arañas (Araneae), este tipo de ataque hasta ahora se ha documentado únicamente en la familia palpimanoide Mecysmaucheniidae, pero también se ha observado una morfología similar en la subfamilia de araneoides Pararchaeinae, un grupo lejanamente relacionado, lo que lleva a la especulación de un ataque de mandíbula trampa en ese linaje también. En este trabajo, usando videos de alta velocidad, evaluamos si la potencia del golpe del quelícero sugiere movimientos impulsados ​​por accionamientos elasticos en la pararqueína *Pararchaea alba*. La velocidad de ataque que alcanzó *P. alba* se situó entre atacantes moderadamente rápidos superando a los mecysmaucheniides más lentos, pero fallando el alcance de los atacantes de alta velocidad más extremos que cuentan con mecanismos de accionamiento elástico. Utilizando micro-tomografía computarizada, comparamos la morfología de los quelíceros de *P. alba* en las posiciones abierta y de reposo, y su musculatura relacionada, y con base en estos resultados proponemos un mecanismo para la función de golpe queliceral que incluye un mecanismo de enganche de inversión de torque. Similar a las arañas mecysmaucheniides de mandíbula trampa, parientes lejanos, la morfología inusual del prosoma en *P. alba* aparentemente permite quelíceros altamente maniobrables con una abertura mucho más amplia que las arañas típicas, lo que sugiere que las articulaciones con mayor maniobrabilidad junto con un mecanismo de enganche, pueden servir como un precursor de movimientos impulsados ​​por accionamientos elásticos.
