# Supplementary figures and images for: Not So Fast: Strike Kinematics of the Araneoid Trap-Jaw Spider Pararchaea alba (Malkaridae: Pararchaeinae)
Source: Integr Org Biol. 2021 Oct 13;3(1):obab027. doi: 10.1093/iob/obab027 (PMC8514421; doi:10.1093/iob/obab027)

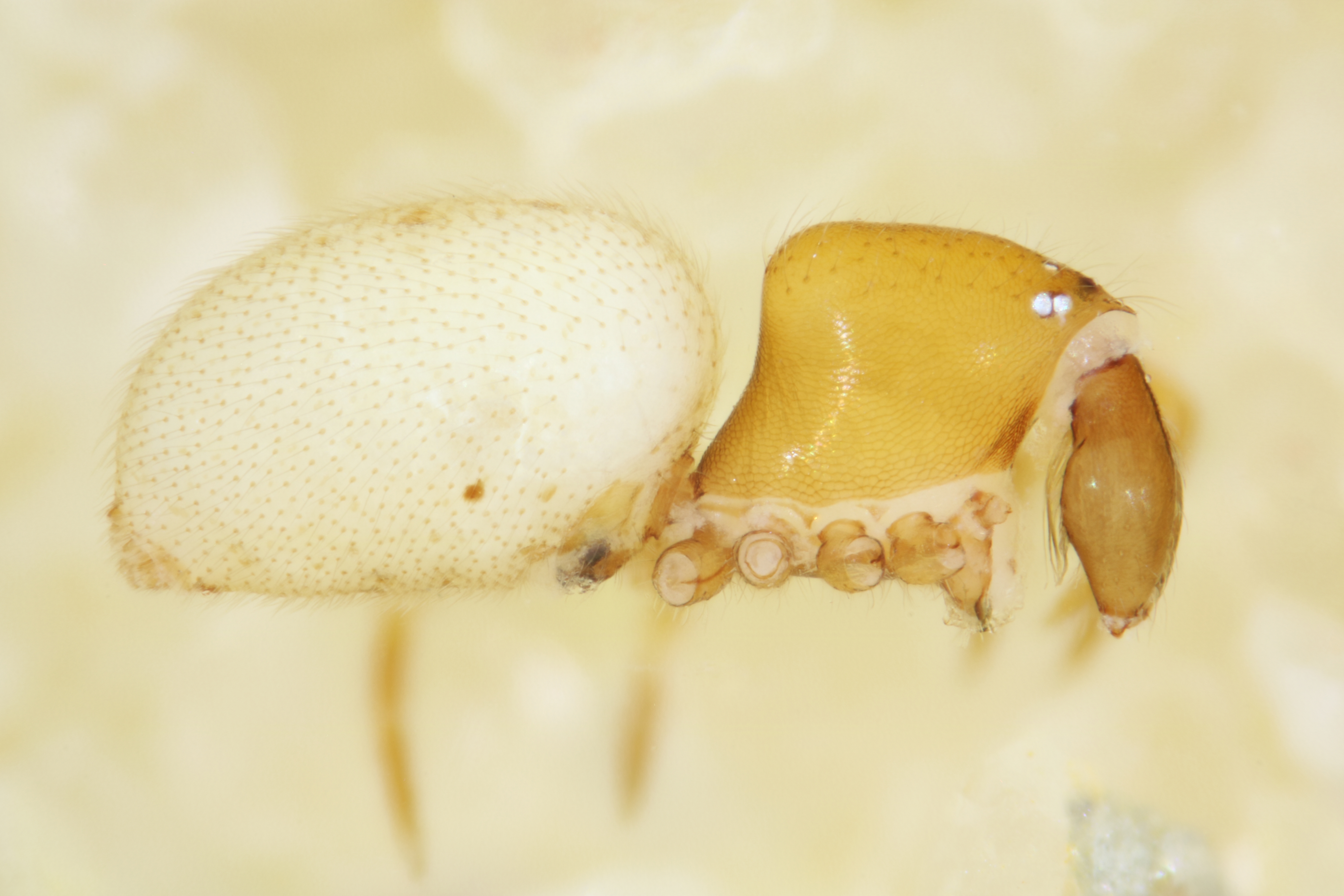

Supplement: obab027_Supplemental_Files [file obab027_supplemental_files.zip › S1_pararchaea_alba_F_CASENT9034330.jpg]
